# Proceedings of the Medical Association of the State of Alabama

**Published:** 1853-07

**Authors:** 


*Proceedings of the Medical Association of the State of Alabama*, Sixth Annual Meeting, December. 1852.

THIS volume of 168 pages was received some time since, and should have received an earlier notice.

The published transactions of our medical organizations have now become an important part of our medical literature; embodying a more reliable account of the character of diseases in different sections of the country, and the various modes of treatment adopted, than can be found elsewhere. Our brethren in Alabama seem to have had a pleasant and profitable meeting. Several interesting papers were read, and considerable time very properly spent in the discussion of the nature and treatment of *Typhoid Fever*. It is known to most of our readers, that several eminent physicians in the Southern States have declared quinine to be scarcely less valuable and efficient in the treatment of typhoid fever, than in fevers of the true periodical type. On this point we find the following recorded in the minutes before us, viz:

Dr. English requested the views of the Fellows of the Association on the use of Quinine in Typhoid Fever. Previous to the present year he had tested its efficacy and at one time was in favor of it, thought it might even cut short the disease. He now considered those opinions erroneous, the abortive treatment with him now failed entirely; has given it in large doses, ten, twelve and fifteen grain doses every three hours, and continued it for thirty-six hours: thought that while the patients were under the influence of those doses, that the pulse became fuller, softer, and less frequent with a free perspiration, but as soon as the medicine was discontinued and its influence passed off, the fever assumed its original features. Had not tried it in only one or two cases, but in several, so that he might be satisfied with the result. He may not have given it in doses large enough, and would like to hear some opinion on the subject.

Dr. Lopez wished Dr. English to state what untoward symptoms he had noticed from the treatment with Quinine.

Dr. English replied that it changed a perspiring skin to a dry and harsh one, and produced derangement of the nervous system. It was very hard to define what was really Typhoid, and the difference at first between that Fever and Remittent was difficult to distinguish; he has sometimes used Quinine as a test between the two, considering it a specific in Remittent Fever; when given in those large doses in Typhoid, thought it did no good; in small doses there was not much alteration.

Dr. Lopez enquired whether the organs of animal life were effected.

Dr. English thought it did appear to produce some narcotic effect. Thinks that one difference between the fever in the beginning, is the absence of pain or complaint on the part of the patient; knows, however, of no distinctive mark by which it can be at first diagnosed.

Dr. Batchelor enquired, if for the first few days he did not find distinct remissions.

Dr. English answered, he did not.

Dr. Reese, of Selma, stated that the difference depended in a great measure on difference in locality. He considered Typhoid Fever as decidedly an enteric disease, and any medicine that would have an irritant effect upon the mucous surfaces pernicious. He considered Calomel poisonous in the treatment of this fever. Saw the disease first in Lowndes county in 1846, where it was endemic; considers it a distinct disease and infectious when malignant; thinks the pathology varies in localities, and this has led to the unsettled opinions in the minds of Physicians, proscribes every thing which would aggravate the enteric disease. Differs with Dr. English even as to the apparent good effect of Quinine, thinks it decidedly pernicious; does not think Quinine produces diaphoresis; checks all secretions.

Dr. Batchelor enquired what were the symptoms when even pain of the abdomen was absent, by which he could at first determine his diagnosis.

Dr. Lopez asked whether Dr. Reese had made many post-mortem examinations, and if he believed the enteric disease to consist of the degeneration of glands spoken of by the authors.

Dr. Reese replied that he had not enjoyed many occasions for post-mortem examinations. He proceeded to mention several symptoms by which he thought the disease could be diagnosed; one symptom was the presence of abdominal heat, recognizable by the touch; another borborygma; another the effect of remedies; if the disease respond to purgatives, most always the purgation is extreme, a small dose of oil often producing a large number of evacuations; another symptom was the odor of fæces in the breath; has noticed this symptom often, and by it been able to recognize the disease; accounts for it by the glands not eliminating, and the odor is exhaled with the breath; another symptom is the tympanities; another cerebral excitement or depression; another hard and contracted state of the abdominal muscles.

Dr. Bates, of Marion, thought there was a great difference of opinion as to what Typhoid Fever is—our best authors have not given a clear description as it appears here. Watson classes it as an axanthematous disease. Dickson ranks it among continued fevers. The question is, what is Typhoid Fever, and what its treatment?

The symptoms, he thinks, are these: Not much complaint at first on the part of the patient, slight pain in the head and back, a sense of weariness; on close examination pulse will be found to vary from 78 to 88; heat, in most cases, increased, especially in the abdomen; sometimes, but not always, slight pain on pressure, and often an uneasiness; evacuations almost healthy, but close examination will discover an unnatural odor, and a lighter color In some cases he has seen the pulse decrease; in three cases which come under his care, it came down to 45; in mild cases the tongue little furred, with the edges a little red, appetite tolerable good; in severe cases these symptoms, generally yet worse, more headache with restlessness at night, often troubled with unpleasant dreams. Considers it an enteric disease, affecting the bowels with degeneration of glands; believes it at first a state of nervous depression which acts upon the glands of the abdomen, and this again by reflex action upon the nervous system. Treatment should correspond; gives nothing which will irritate, food nutritious, demulcent drink, and bowels kept open by laxatives of the mildest nature. His favorite prescription is Chlorate Potass. as a mild, cooling refrigerant, which greatly relieves the patient of his thirst and dry tongue.

Believes mercurials given for their constitutional effects, injurious. Gives it some times in its mildest form as Blue Mass, when the liver seems to be a little torpid, or in the form of Hydg. Cum Creta, when diarrhœa supervenes.

When the system becomes depressed with sub-sultus tendinum, dry and harsh tongue, he used stimulants, and for this purpose prefers the Carb. Ammoniæ, of which he thinks very highly. He however, endeavors to anticipate this stage by giving the Carb. Ammoniæ in small doses. With regard to Quinine, he at first gave it experimentally in 2 or 3 grain doses every two or three hours, and found it produced exaltation of the vital forces. Has given it in ten grain doses every three or four hours with same effect. Have never seen it do any good unless there was an exacerbation, and has then given it in 10 or 15 grain doses to relieve the exacerbation, but never found it stop the progress of the disease.

Dr. Gee replied what were the symptoms for the first five days.

Dr. Baaes required there was restlessness, with pain in the head and back, pulse either higher or lower. In the morning he noticed the pulse was in most cases about 78, but at night rose to 88.

Dr. Lopez enquired whether he thought it a paroxysmal disease.

Dr. Bates replied he did not. Believed it to be an ataxic or adynamic disease, *sui generis.* Thinks Remittent Fever, badly treated, may run into a low form of fever, and that Typhoid Fever may be modified by a malarial influence.

Dr. Jenkins, of Wilcox, had seen in a great majority of the cases which had come under his notice a distinct remission from 3 to 5 in the morning. In nearly all his cases there was diffuse diaphoresis, and the disease was infectious. He had used Iron, Quinine and Mercury separately, but with no beneficial effect. He had at last adopted the expectant mode of treatment with success.

The President enquired whether there was any eruption visible.

Dr. Jenkins replied, that he had seen in a majority of cases sudamina on the breast, and in some few, rose colored spots on the abdomen.

Dr. Bates has never been fully satisfied that he had seen any eruption. Cases were mostly negroes, and he examined very closely.

Dr. Marks, of Selma, thought in the state of depression Quinine the best stimulant, but had never seen it cut short the disease.

Dr. Fair, of Selma, stated, that as far as his experience went, he had never seen Quinine produce any curative effect in this disease. He thinks Typhoid a special and peculiar disease, and that it acts primarily on the nervous system. Dr. F. spoke at some length, but the Secretary not having the benefit of Dr. F.'s correction of his notes, has thought it best, for fear of misstating some of the Dr's. opinions, not to publish them.

It will be seen that the opinions here expressed, do not give countenance to the idea that quinine is capable of exerting any curative influence over typhoid fever, and such, we think, will finally be the unanimous verdict of the profession both in the North and the South.

The following are the essays and papers contained in the volume before us, viz: Report on Indigenous Botany, by A. Denny, M.D.; Report on Diseases of Centreville and Vicinity, by J. W. Crawford, M.D.; Report on Surgery, by L. H. Anderson, M.D.; Cases reported by J. F. Gee, M.D.; Report on the article Gelseminum Sempervirens, by J. C. Batchelor, M.D.; Report on Diseases of Cahaba, by J. A. English, M.D.; Report an Indigenous Botany of Perry, by F. A. Bates, M.D.; Changeability of Disease, by W. Taylor, M.D.; Paper on the Unity of Disease, by H. Backus, M.D.; Prize Essay on the Summer and Autumnal Fevers of South Alabama, to which is appended some remarks on the Diagnosis and treatment of Typhoid Fever, by L. H. Anderson, M.D.; Valedictory Address, by F. A. Bates, M.D.; Reports on the Number and Character, etc, of Practitioners of Medicine, by J. P. Barnes, M.D., A. Denny, M.D., F. E. Gordon, M.D., and R. H. Irwin, M.D.

Some of these papers are of sufficient importance to require a separate and more extended notice. Such is particularly the case in reference to the Prize Essay of Dr. Anderson, which (if time permits) we will examine more in detail in the next number of the Journal. N. S. D.

